# Screening for Distress and Health Outcomes in Head and Neck Cancer

**DOI:** 10.3390/curroncol29060304

**Published:** 2022-05-24

**Authors:** Bryan Gascon, Aliza A. Panjwani, Olivia Mazzurco, Madeline Li

**Affiliations:** 1 Temerty Faculty of Medicine, University of Toronto, Toronto, ON M5S 1A8, Canada; bryan.gascon@uhnresearch.ca; 2 Department of Supportive Care, Princess Margaret Cancer Centre, University Health Network, Toronto, ON M5G 2C1, Canada; aliza.panjwani@uhnresearch.ca (A.A.P.); olivia.mazzurco@uhnresearch.ca (O.M.); 3 Department of Psychiatry, University of Toronto, Toronto, ON M5T 1R8, Canada; 4 Institute of Medical Science, University of Toronto, Toronto, ON M5S 1A8, Canada

**Keywords:** head and neck cancer, distress screening, depression, anxiety, emotional distress, survival, ESAS, MDASI, PHQ-9, GAD-7

## Abstract

Head and neck cancers (HNC) have higher rates of emotional distress than other cancer types and the general population. This paper compares the prevalence of emotional distress in HNC across various distress screening measures and examines whether significant distress or distress screening are associated with cancer-related survival. A retrospective observational cohort design was employed, with data collected from the Distress Assessment and Response Tool (DART) and linkages to administrative databases from 2010 to 2016. Descriptive and prevalence data were reported using multiple concurrently administered distress tools, including the Patient Health Questionaire-9 (PHQ-9), Generalized Anxiety Disorders-7 (GAD-7), Edmonton Symptom Assessment Scale-revised (ESAS-r), and MD Anderson Symptom Index-Head and Neck module (MDASI-HN). Across measures, 7.8 to 28.1% of the sample reported clinically significant emotional distress, with PHQ-9 and GAD-7 identifying lowest prevalence of moderate/severe distress, and the ultrashort distress screens within ESAS-r and MDASI-HN performing equivalently. Cox hazards models were used in univariate and multivariate survival analyses. ESAS depression (≥4), but not anxiety, was associated with increased risk of cancer-related mortality and patient completion of DART was associated with greater cancer-related survival. The findings underscore the importance of implementing routine distress screening for HNC populations and the utility of ultra-brief screening measures.

## 1. Introduction

Patients with head and neck cancers (HNC) report higher levels of distress [1] and rates of suicide [2] than other cancers. While recent advancements in surgery, radiation, chemotherapy, immunotherapy, and multimodal approaches have increased survival for HNC patients [3,4,5], curative treatment must balance increased survival with potentially decreased quality of life from treatment sequelae. Disfigurement, limited shoulder and neck mobility, pain, fatigue, and impairment of swallowing, chewing, and speech functions are some of the symptoms impacting health-related quality of life and emotional distress among HNC survivors [6,7]. Such distress has been associated with treatment noncompliance, increased healthcare costs, delays in seeking treatment, and decreased survival [8,9,10,11].

Emotional distress, considered the sixth vital sign in cancer care, is a broad term that has been defined by the National Comprehensive Cancer Network as “a multifactorial unpleasant experience of a psychological (i.e., cognitive, behavioral, emotional), social, spiritual, and/or physical nature that may interfere with one’s ability to cope effectively with cancer and its physical symptoms and treatment. Distress extends along a continuum, ranging from common, normal feelings of vulnerability, sadness, and fears, to problems that can become disabling, such as depression, anxiety, panic, social isolation, and existential and spiritual crisis” [12]. Standardized screening for distress refers to the use of standardized measures to quantitatively assess distress in clinical settings. It facilitates increased detection of emotional distress, allowing health care teams to provide appropriate psychosocial care to improve both psychosocial and health outcomes. Various distress screening tools have been used to describe the prevalence of distress in the HNC patient population, although there is no consensus on which measure demonstrates the best psychometric properties to inform clinical utility. 

Using the brief, rapidly administered distress thermometer (DT), many studies have demonstrated prevalence rates of clinically significant distress from 25–60% for HNC patients across the disease trajectory [13,14,15,16,17,18]. In comparison, 15–51% of HNC patients report moderate to severe emotional distress using items from longer and more comprehensive screening tools, such as the Edmonton Symptom Assessment Scale (ESAS) [19,20], MD Anderson Symptom Inventory (MDASI) [21,22], and the University of Washington Head and Neck Cancer Questionnaire (UW-QOL V4) [23]. Studies using the Patient Health Questionnaire-9 (PHQ-9) and Generalized Anxiety Disorder Scale-7 (GAD-7), which map onto symptomology from the Diagnostic and Statistical Manual of Mental Health Disorders, Fifth Edition (DSM-5), have found that 8–21% report clinically significant symptoms [24,25,26]. Levels of distress are typically highest during the treatment phase [14,20,27] and may persist for years post-treatment for a considerable proportion of survivors, owing to financial impacts and social withdrawal resulting from disfigurement and functional deficits [28].

However, there have been few comparative studies to guide the selection of optimal distress screening tools in HNC, and uptake of distress screening has been limited by the lack of hard health outcomes associated with distress screening. The distress assessment and response tool (DART) is a comprehensive screening system, developed and implemented at the Princess Margaret Cancer Centre (PM) in Toronto, Canada as a routine standard of care for all patients with cancer. DART contains specific distress measures, such as the PHQ-9 [29] for depression, the GAD-7 for anxiety [30], as well as several broad cancer distress burden instruments, including the Edmonton Symptom Assessment System-revised (ESAS-r) [31] and the MD Anderson Symptom Inventory Head and Neck module (MDASI-HN) [21], which contain items assessing emotional distress.

DART provides a unique opportunity to examine the utility of multiple, concurrently administered distress screening tools in a large longitudinally-followed cohort of patients with HNC. Consequently, in a heterogenous population of HNC patients completing DART at diagnosis, this study aimed to (1) compare the prevalence of distress across multiple distress screening measures; (2) examine the association between clinically significant emotional distress and cancer-related survival; and (3) explore the relationship between completion of distress screening and cancer-related survival.

## 2. Materials and Methods

### 2.1. Study Design and Setting

This is a retrospective observational study based on administrative and clinical data routinely collected at PM. The study cohort consisted of adult patients (ages 18 years or older) with a pathologically-confirmed malignant head and neck cancer diagnosis treated at PM between 1 January 2010 and 31 December 2016. Patients with tumors of uncertain behavior or of in situ nature (non-malignant cases) were excluded from the analyses. Research ethics board (REB) approval was obtained from the University Health Network to use the administrative and clinical data reported in this paper.

### 2.2. Distress Assessment and Response Tool (DART)

In addition to the PHQ-9, GAD-7, ESAS-r, and MDASI-HN, DART includes the following measures: Eastern Cooperative Oncology Group functional status (ECOG), the Social Difficulties Inventory (SDI-21) [32], Informational and Spiritual Problems domains of the Canadian Problem Checklist (CPC), questions about interest in smoking cessation, distress risk factors (living situation and psychiatric history), and desire for support [33]. The PHQ-9 is a 9-item scale that is concordant with the DSM-5 criteria for major depression, with responses to each item ranging from “0-not at all” to “3-nearly every day”, for a total range of 0–27 [29]. The GAD-7 is a 7-item scale for generalized anxiety disorder, scored on a similar 0–3 point scale, with a total range of 0–21 [30]. The ESAS-r comprises nine symptoms commonly experienced by patients with cancer: pain, tiredness, nausea, depression, anxiety, drowsiness, appetite, well-being, and shortness of breath. The severity of each symptom at the time of assessment is rated on a numerical scale from “0-symptom is absent” to “10-worst possible severity” [31]. The MDASI-HN is a 28-item multi-dimensional measure of general cancer-related and HNC-specific symptoms in the last 24 h, each scored on a similar 0–10 point scale with separate sub-scales for symptom severity and functional interference [21].

DART was implemented at PM in 2010 in a step-wise fashion, incorporating intelligent programming to minimize survey burden and customizing additional screening measures in individual cancer sites over time [33]. Prior to March 2013, all DART measures were administered in tandem. After March 2013, DART adopted computer adaptive testing, which involved screening with full patient-reported outcome measures based on pre-screening, using validated cut-scores on ultrashort measures [34]. The PHQ-9 was then only administered if patients report ≥2 on the ESAS-r depression item (ESAS-D), and GAD-7 only administered if patients report ≥3 on the ESAS-r anxiety item (ESAS-A). In 2014, the MDASI-HN module was added to DART in all HNC clinics, programmed on alternate visits with the ESAS-r.

Upon patient completion of DART, personalized reports summarizing screening results are generated and uploaded to the patients’ electronic medical record for review by oncology nurses or physicians. Patients with subthreshold distress are directed to self-management and psychoeducational resources, while patients with moderate to severe levels of distress are assessed and managed according to the stepped care model of supportive care described in national distress management guidelines in Canada [35].

### 2.3. Data Sources

Data to achieve Aims 1 to 3 were obtained from several sources and from different time periods, based on DART programming considerations and cause-of-death data availability. For Aim 1 (comparing the prevalence of distress across multiple distress screening measures), data on distress prevalence at diagnosis was extracted from the PM DART research database. Two different HNC patient populations were analyzed to enable comparisons across concurrently completed distress measures to avoid confounding from the computer adaption beginning in March 2013. To compare ESAS-D vs. PHQ-9 and ESAS-A vs. GAD-7, the first DART survey completed by patients within 3 months of diagnosis between January 2010 and March 2013 was extracted for analysis. To compare ESAS-A, ESAS-D, MDASI-distress, MDASI-sadness, and MDASI-depression-mood component scores [36], the first DART survey completed by patients within 3 months of diagnosis between January 2014 and December 2016 was extracted for analysis.

For the survival analyses in Aim 2 (examining the association between clinically significant emotional distress and cancer-related survival) and Aim 3 (exploring the relationship between completion of distress screening and cancer-related survival), data on causes-of-death and death dates (available only up to 31 December 2014) were obtained via linkage to the provincial Ontario Health administrative database, which houses death certificates of all deceased Ontario residents diagnosed with cancer from the Registrar General of Ontario [37]. HNC patients were linked to the PM Cancer Registry to extract the following available data elements: age, sex, marital status, cancer type, cancer stage, and postal codes. Postal codes were linked to census data made available by Statistics Canada to approximate patient’s median household income [38]. Patient-level DART completion status and distress levels were obtained via linkage to the PM DART research database. Descriptive data on psychosocial and palliative care visits among the study cohort were obtained via a link to the PM data warehouse.

### 2.4. Data Analytic Strategy

For descriptive analyses in Aim 1, proportions of patients meeting none/mild, moderate, and severe thresholds on each individual measure were compared using chi-square tests. For ESAS-D and ESAS-A, none/mild, moderate, and severe were defined as ≤3, 4–6, and ≥7 [35,39], respectively. For PHQ-9, none/mild, moderate, moderately severe/severe depressive symptoms were defined as ≤9, 10–14, and ≥15 [29], respectively. For GAD-7, none/mild, moderate, and moderately severe/severe anxiety were defined as ≤9, 10–14, and ≥15 [30], respectively. For MDASI-sadness, and MDASI-distress moderate/severe symptoms were defined as ≥4 [36]. For MDASI-depressive-mood component score (sum of MDASI-sadness, fatigue, mood, relations with others, and enjoyment of life), moderate/severe depressive symptoms were defined as ≥19 [36].

For survival analyses in Aim 2, consecutive HNC patients who completed DART within 3 months of diagnosis between 1 January 2010 to 31 December 2013 were included. Cumulative incidence curves were calculated to separately estimate the univariate association between moderate/severe (ESAS-D/A ≥4) depression and anxiety reported at diagnosis and cancer-related survival (up to 31 December 2014), with deaths by noncancer-related causes treated as a competing risk. To estimate a multivariable association, propensity scores for depressive and anxiety symptoms were calculated using a logistic regression model that included the following variables: age, sex, number of malignancies, cancer types, cancer stage, marital status, and median household income. Computed propensity scores were then incorporated into inverse probability of treatment weighting analyses (IPTW) using a Cox hazards model to estimate hazard ratios (HR), with 95% confidence intervals (CI) constructed using bootstrap resampling.

For Aim 3, consecutive HNC patients seen at PM between 1 January 2010 and 31 December 2013 were included in analyses. Similar to Aim 2, survival analyses were conducted to assess the association between DART completion status and cancer-related survival (up to 31 December 2014). To assess the robustness of our findings, we performed alternative statistical approaches, including stratification on propensity score, multivariable Cox-regression and Fine and Grey regression (unweighted). Descriptive statistics were provided for cohorts analyzed in Aim 2 and 3. Differences in baseline characteristics among DART completers vs. non-completers were compared with Wilcoxon or chi-square tests for continuous and categorical variables, respectively. Gray’s test was used to compare differences between cause-specific cumulative incidence curves of each analysis group.

## 3. Results

### 3.1. Prevalence of Distress across Patient-Reported Outcome Measures

Between January 2010 and March 2013, 347 patients with HNC completed DART within 3 months of diagnosis. Among this cohort, 266 (76.7%) were male, 252 (72.6%) were diagnosed at stage 3 or 4, and the mean age of the cohort was 60.7 years (standard deviation [SD]: 12.3; range: 18–91 years). Table 1 compares the distribution of depressive and anxiety symptom severity across ESAS-D vs. PHQ-9 and ESAS-A vs. GAD-7, respectively. A significantly greater proportion of moderate-severe depression was reported on ESAS-D item compared to PHQ-9 (*p* < 0.05). For anxiety, significantly greater proportions of moderate-severe anxiety was reported on ESAS-A item compared to GAD-7 (*p* < 0.05).

Table 2 compares the distribution of distress symptom severity across ESAS-A, ESAS-D, MDASI-distress, MDASI-sadness, and MDASI-HN mood component scores completed between 2014 and 2016. During this timeframe, 356 patients with HNC completed these measures through DART within 3 months of diagnosis. Among this cohort, 260 (73.0%) were male, 232 (65.1%) were diagnosed at stage 3 or 4, and the mean age of the cohort was 57.8 years (standard deviation [SD]: 11.1; range: 28–93 years). A significantly greater proportion of moderate/severe depressive symptoms were reported on MDASI-depression mood component score compared to other ESAS-r and MDASI-HN items (*p* < 0.05).

### 3.2. Emotional Distress and Cancer-Related Survival

**Figure 1:**
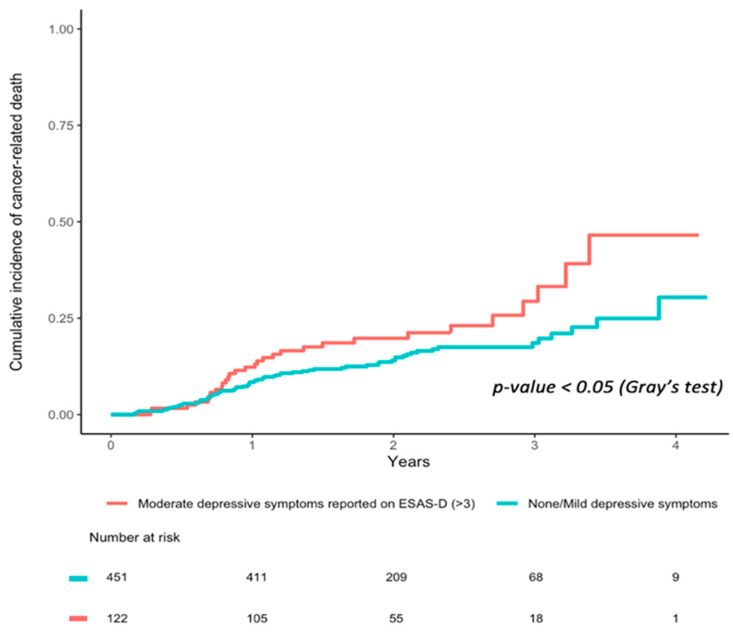
Cumulative incidence of cancer-related death in patients with moderate/severe vs. none/mild ESAS depressive symptoms reported at diagnosis.

Table 3 show the baseline characteristics of the Aim 2 study population (n = 573). Univariate cumulative incidence curves for cancer-related death in those reporting moderate/severe vs. none/mild ESAS depression item at diagnosis are plotted in Figure 1. Over the first five years of follow-up (up to 31 December 2014), cancer-related survival was significantly higher in those who reported moderate/severe ESAS depression compared to patients that did not (Gray’s test, *p* < 0.001).

In our multivariable propensity score-weighted analyses accounting for cancer stage, sex, household income, and marital status, moderate/severe depression at diagnosis was associated with higher risk of cancer-related death compared to those without depressive symptoms (Table 4; IPTW hazard ratio (HR) 1.66; 95% CI 1.47–1.86). An alternative statistical model, stratification on propensity score (sPS) yielded consistent results, with similar magnitudes of this association to those derived from our main IPTW analyses.

**Figure 2:**
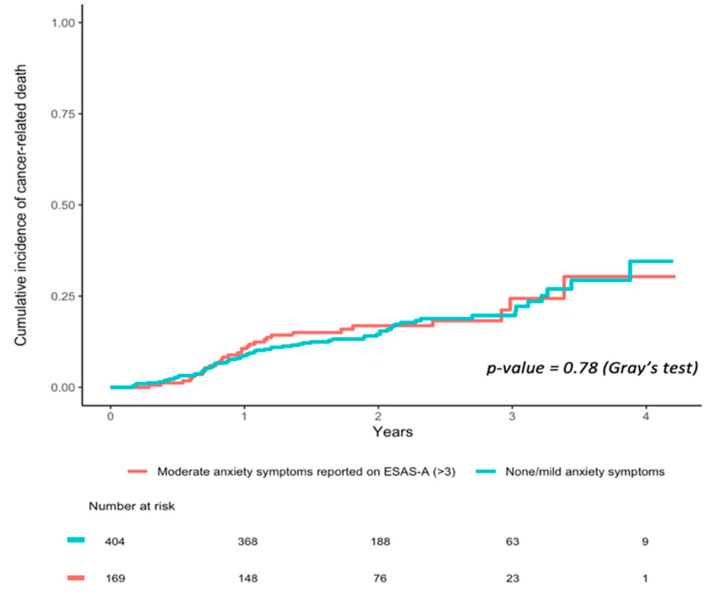
Cumulative incidence of cancer-related death in patients with moderate/severe vs. none/mild ESAS anxiety symptoms reported at diagnosis.

Univariate cumulative incidence curves for cancer-related death in those reporting moderate/severe vs. none/mild ESAS anxiety item at diagnosis are plotted in Figure 2 Over the first five years of follow-up (2010–2014), cancer-related survival was no different in those who reported moderate/severe ESAS anxiety compared to patients who did not (Gray’s test, *p* = 0.78).

In our multivariable propensity score-weighted analyses accounting for cancer stage, sex, household income, and marital status, moderate/severe anxiety at diagnosis was not associated with cancer-related survival (Table 5; IPTW hazard ratio (HR) 1.03; 95% CI 0.88–1.19).

### 3.3. Distress Screening Completion and Cancer-Related Survival

Table 6 shows the baseline characteristics of Aim 3 study population, stratified by DART completion status. Of 2628 eligible HNC patients, 1418 (54.0%) completed DART at least once from 2010 to 2014 and were included in the survival analysis. Patients who completed DART were more likely to be younger, male, have later stage disease, and higher household income than patients who never completed DART. Patients in the DART completer group had a greater proportion of psychiatry and psychology visits (*p* < 0.001) but a comparable number of visits to palliative care (*p* = 0.29) than those who did not complete DART.

**Figure 3:**
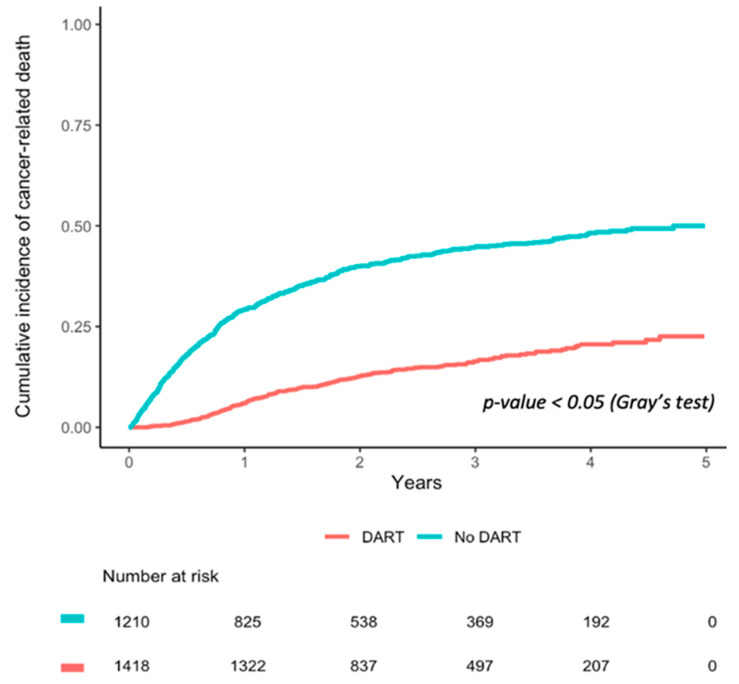
Cumulative incidence of cancer-related death in DART completers vs. non-completers.

Univariate cumulative incidence curves for cancer-related death in DART completers and non-completers are plotted in Figure 3 Over the first five years of follow-up (2010–2014), cancer-related survival was significantly higher in those who completed DART compared to those that did not (Gray’s test, *p* < 0.05).

In our multivariable propensity score-weighted analyses accounting for cancer stage, sex, household income, and marital status, DART completion was associated with significantly lower risk of cancer-related death than DART non-completers (Table 7; IPTW hazard ratio (HR) 0.23; 95% CI 0.22–0.25).

## 4. Discussion

The purpose of the current study was to describe the prevalence of distress using multiple screening tools among patients within three months of HNC diagnosis. We also examined whether the presence of clinically significant distress at diagnosis and completion of comprehensive distress screening was associated with cancer-related survival.

Comparing the different distress screening tools in our sample, we found the prevalence of moderate/severe depressive and anxiety symptoms were lowest when examined using the PHQ-9 (11.5%) and GAD-7 (7.8%). These numbers are lower compared to other studies of HNC using the same measures [40,41], though these other studies tended to consist of patients in survivorship. In contrast, the MDASI depression-mood component captured the highest number patients with clinically significant depressive symptoms (28.1%). This finding makes intuitive sense, as the MDASI depression-mood component consisted of five items tapping into critical depressive symptoms (i.e., mood, anhedonia, (dis)connection; [36]) and, thus, may provide more sensitivity than single item scales, but less specificity than longer measures of anxiety or depression, such as the PHQ-9 [36]. The single items assessing mood, ESAS-A (18.8%), ESAS-D (20.0%), MDASI-distress (25.0%), MDASI-sadness (18.8%), all captured similar levels of distress, consistent with psychometric studies evidencing strong concurrent validity in ultrashort measures, but low positive predictive value [42].

Further supporting the use of distress screening tools at cancer centers, our data found that clinically significant depressive symptoms predicted lower survival, even after the inclusion of cancer stage, sex, household income, and marital status as covariates. These findings are in line with studies demonstrating a link between psychological comorbidities and cancer survival and mortality [43,44]. A review pooling unpublished participant data from 16 community-based prospective cohort studies in England and Scotland found that distress predicted cancer-related mortality; this association was robust for several cancers [45]. Moreover, a recent meta-analysis provided support for the notion that overall clinically diagnosed anxiety and depression, as well as emotional distress assessed by symptom scales, was related to poor survival in cancer patients; site specific analyses demonstrated that this significant association was limited to patients with lung cancer [46]. Although this meta-analysis included a catch-all category of “all cancers”, it did not appear to include HNCs as a specific site [46]. Our findings help fill this gap for patients with HNCs.

In contrast to the finding for depression, anxiety was unrelated to patient survival. This disparity, wherein depression has stronger associations with survival and anxiety has null effects, has also been reported by several studies [44,47,48]. Meta-analytic estimates have shown that depression, as well as comorbid depression and anxiety, are associated with survival in breast cancer, but anxiety alone is not [49]. Although general cancer-specific anxiety and the anxiety unique to living with HNC may add to the emotional burden experienced by these patients, it may not necessarily contribute to poorer survival. Furthermore, while anxiety appears to decrease from pre- to post-treatment, depression has been shown to increase post-treatment and may remain a more chronic vulnerability factor in HNCs [50].

Depression is also closely linked to suicide [51] and desire for hastened death [52], and thus may be a far more relevant index of distress pertaining to both overall and cancer-specific survival than anxiety, particularly in HNC. Patients with HNCs are two times more likely to commit suicide than patients with other cancers and four times more likely compared to the general population [53]. Compared to anxiety, depression also has stronger negative effects on treatment adherence, with meta-analytic estimates indicating that depressed individuals are three times more likely to be treatment noncompliant than their non-depressed counterparts [54].

Our study also found that HNC patients who completed DART had a significantly lower risk of cancer-related mortality than those who did not complete DART. This finding remained significant even after accounting for age, sex, cancer stage, household income, and marital status; though not all factors which may affect an individual’s likelihood of completing distress screening could be accounted for. As such, although an intriguing finding, causal relationships cannot be concluded. It may be that completing DART is associated with enhanced symptom management and reduced distress. However, it is equally possible that those who participate in distress screening are inherently a lower risk population and, thus more willing to engage in positive health behaviors. However, the distress screening literature has clearly demonstrated benefits for other health outcomes, including identification of individuals in need of psychosocial or palliative services, improved satisfaction with care, increased patient–physician communication, and decreased healthcare utilization [33,55,56].

### 4.1. Clinical Implications

Despite evidence that significant distress is associated with negative health outcomes and screening for distress is associated with improved emotional well-being and fewer physical and practical concerns in previous HNC research [8], the uptake of distress screening in institutions remains limited. Improving health outcomes by screening for emotional distress requires attention to best practices from implementation science research [57]. Since positive screening must be followed by effective psychosocial care, an institution’s clinical capacity may determine which screening tool is optimal. Our results suggest that longer measures such as PHQ-9 and GAD-7 result in lower volumes of positive screens with higher specificity. In contrast, ultrashort tools within a comprehensive symptom screening measure, such as ESAS-r or MDASI-HN, may reduce the screening burden, while efficiently capturing a range of symptoms that may contribute to distress. Ultrashort screening measures have demonstrated validity [58], but as with any screening test, a positive screen only identifies distress and must be followed by a clinical assessment to determine the need for an intervention. Ultimately, the selection of an optimal distress screening tool and screening cut-scores depends on institutional factors, such as capacity and availability of psychosocial resources and other symptom management priorities. Given that the MDASI-HN is specific to HNC symptoms, and as our results demonstrated the equivalence to ESAS-r in capturing emotional distress, we recommend the use of MDASI-HN for assessing symptom burden in the HNC population. However, the choice of distress screening measure is perhaps less important than ensuring an adequate clinical response to positive screens.

Lack of engagement of both front-line clinical teams and patients in follow-up to positive distress screens is the main reason few patients receive adequate supportive care [18,59,60]. Evidence that distress and screening for distress is associated with hard outcomes such as survival, may make distress screening more meaningful to front-line clinicians. Other research has found that of the individuals who received a screen-based referral to psychosocial services, 17% declined services. Of those who went on to complete an assessment with a psychosocial provider, only 19% completed at least one follow-up appointment [61]. Future research may benefit from examining patient-related barriers to acceptance of psychosocial services. One solution to remove burden from patients and staff alike may be implementing a stepped care approach to both screening [33] and psychosocial intervention [62,63,64].

### 4.2. Limitations

Strengths of the current study include the analysis of multiple concurrently administered validated screening tools and health outcomes collected in a real-world clinical setting. Limitations include the inability to analyze more specific measures of depression and anxiety (i.e., PHQ-9, GAD-7 and relevant MDASI items) in survival analyses, as changes in DART programming resulted in sample sizes that were underpowered for survival analyses. In addition, while we included several covariates (i.e., age, sex, cancer stage, income, and marital status) in our multivariate analyses, we did not have race or ethnicity data. The data on racial health disparities in HNC in North America are varied and often based on studies with relatively small sample sizes. For example, research has shown that African Americans with HNC have decreased survival rates compared to white patients and that the former present with more advanced disease at diagnosis [65]. When receiving multidisciplinary care, the difference in survival is not significant, although racial disparities in treatment regimen may remain [65]. In Ontario, Canada, all-cause mortality for immigrants and specifically Chinese people in HNC was lower compared to South Asians and other matched controls [66]. Future work in large cohort studies should incorporate race/ethnicity data, as this will allow for meaningful identification of vulnerable subgroups. The lack of data on other medical and social determinants of survival (e.g., living situation, pre-existing psychiatric conditions, medical comorbidities) is another important limitation, as these factors may affect both the ability and willingness to complete DART and survival outcomes. Therefore, causal relationships between distress screening, distress, and survival in HNC cannot be concluded.

## 5. Conclusions

HNC patients report disproportionately high levels of emotional distress, which is associated with decreased cancer-related survival. Screening for distress can enable earlier identification of distress for clinicians to provide targeted supportive interventions [67]. Future studies should prospectively examine the relationship between distress screening and cancer-related survival in HNC, focusing on underlying clinical mechanisms or mediators. It is clear that tools and methods for screening for distress in HNC exist, and failure to implement such screening deprives vulnerable patients of the opportunity for health. It has been argued that screening for distress and ensuring appropriate referrals and uptake of psychosocial services after identification of positive screens are critical steps in providing ethical care to patients with HNC [68].

## Figures and Tables

**Table 1:** Distress prevalence at diagnosis across PHQ-9, GAD-7, ESAS-D, and ESAS-A from HNC patients seen at PM between 2010 to March 2013 (n = 347).

Severity	ESAS-D	PHQ-9	*p*-Value
None/Mild	271 (78.1%)	307 (88.5%)	*p* < 0.05
Moderate	49 (14.1%)	22 (6.3%)	
Severe	27 (7.8%)	18 (5.2%) ^a^	
**Severity**	**ESAS-A**	**GAD-7**	***p*-Value**
None/Mild	246 (70.9%)	320 (92.2%)	*p* < 0.05
Moderate	66 (19.0%)	15 (4.3%)	
Severe	35 (10.1%)	12 (3.5%) ^a^	

^a^ Indicates proportion combining moderately severe and severe interpretation categories on measure.

**Table 2:** Distress prevalence at baseline across ESAS-A, ESAS-D, MDASI-distress, MDASI-sadness, and MDASI depression-mood component score between 2014 to 2016.

Severity	ESAS-A	ESAS-D	MDASI-Distress	MDASI-Sadness	MDASI-DM	*p*-Value
None/Mild	289 (81.2%)	285 (80.0%)	267 (75.0%)	289 (81.2%)	256 (71.9%)	*p* < 0.05 ^a^
Moderate/Severe	67 (18.8%)	71 (20.0%)	89 (25.0%)	67 (18.8%)	100 (28.1%)	*p* = 0.13 ^b^

Note. MDASI-DM = MDASI depression-mood component. ^a^ Comparison of distress prevalence between the single item measures (ESAS-A, ESAS-D, MDASI-distress, MDASI-sadness) and MDASI-DM. All single item measures were significantly different from MDASI-DM. ^b^ Comparison of prevalence among single item measures and excluding MDASI-DM.

**Table 3:** Baseline characteristics of Aim 2 study population.

Characteristics	Population (n = 573)
Age at First Contact-Years	
Mean (SD)	60.6 (12.6)
Min and Max	18.9–91.3
SEX—n (%)	
Female	142 (24.8)
Male	431 (75.2)
Cancer Stage—n (%)	
1–2	171 (29.8)
3–4	402 (70.2)
Marital Status—n (%)	
Married/Common-Law	95 (16.6)
Single/Divorced/Widowed	41 (7.2)
Unknown	437 (76.3)
Estimated Household Income—USD	
Mean (SD)	68, 800.710 (28, 361.414)

**Table 4:** Hazard ratios for cancer-related deaths in patients with moderate/severe vs. none/mild on the ESAS depression item at diagnosis.

Method	Estimate	Hazard Ratio	95% CI
IPTW	Naïve	1.65	1.10, 2.53
	Bootstrap	1.66	1.47, 1.86
sPS	Naïve	1.47	0.95, 2.28
	Bootstrap	1.49	1.33, 1.65

Note. IPTW = inverse probability of treatment weighting analyses; sPS = stratification on propensity score.

**Table 5:** Hazard ratios for cancer-related deaths in patients with moderate/severe vs. none/mild ESAS anxiety symptoms at diagnosis.

Method	Estimate	Hazard Ratio	95% CI
IPTW	Naïve	1.04	0.68, 1.60
	Bootstrap	1.03	0.88, 1.19
sPS	Naïve	1.01	0.66, 1.55
	Bootstrap	1.03	0.93, 1.12

Note. IPTW = inverse probability of treatment weighting analyses; sPS = stratification on propensity score.

**Table 6:** Aim 3 cohort baseline characteristics stratified by DART completion status.

Covariate	DART Completed (n = 1418)	No DART (n = 1210)	*p*-Value
Age at First Contact-Years			0.001
Mean (SD)	60.2 (13.4)	64.7 (13.5)	
Min and Max	18.1–97.4	18.0–95.8	
SEX—n (%)			0.001
Female	415 (29.3)	387 (32.0)	
Male	1003 (70.7)	823 (68.0)	
Cancer Stage—n (%)			0.001
1-2	471 (33.2)	367 (30.3)	
3-4	876 (61.8)	595 (49.2)	
Unknown	46 (3.2)	202 (16.7)	
Unstageable	25 (1.8)	46 (3.8)	
Marital Status—n (%)			0.001
Married/Common-Law	234 (16.5)	191 (15.8)	
Single/Divorced/Widowed	108 (7.6)	102 (8.4)	
Unknown	1076 (75.9)	917 (75.8)	
Estimated Household Income-$			0.001
Mean (SD)	68,402.9 (29,687.6)	64,129.7 (28,949.5)	
Seen by Psychiatry and Psychology—n (%)			<0.001
	105 (7.4)	39 (3.2)	
Seen by Palliative Care—n (%)			0.290
	95 (6.7)	94 (7.8)	

**Table 7:** Hazard ratios for cancer-related deaths in DART completers vs. non-completers.

Method	Estimate	Hazard Ratio	95% CI
IPTW	Naïve	0.23	0.20, 0.28
	Bootstrap	0.23	0.22, 0.25
**Sensitivity Analysis Methods**	**Estimate**	**Hazard Ratio**	**95% CI**
Cox Proportional Hazards Model	Univariate	0.23	0.20, 0.27
	Multivariate (adjusted)	0.25	0.21, 0.29
Fine and Gray model	Univariate	0.24	0.21, 0.28
	Multivariate (adjusted)	0.27	0.23, 0.32

IPTW = inverse probability of treatment weighting analyses.

## Data Availability

Not applicable.
